# Intestinal barrier dysfunction mediates Whipple's disease immune reconstitution inflammatory syndrome (IRIS)

**DOI:** 10.1002/iid3.622

**Published:** 2022-04-28

**Authors:** Julian Friebel, Katina Schinnerling, Anika Geelhaar‐Karsch, Kristina Allers, Thomas Schneider, Verena Moos

**Affiliations:** ^1^ Department of Cardiology Charité‐University Medicine Berlin Germany; ^2^ Berlin Institute of Health at Charité‐Universitätsmedizin Berlin, BIH Biomedical Innovation Academy BIH Charité Clinician Scientist Program Berlin Germany; ^3^ Medical Department I, Gastroenterology, Infectious Diseases and Rheumatology Charité‐University Medicine Berlin Germany; ^4^ Present address: Katina Schinnerling, Departamento de Ciencias Biológicas, Facultad de Ciencias de la Vida Universidad Andrés Bello Santiago Chile

**Keywords:** barrier dysfunction, immune reconstitution inflammatory syndrome, inflammation, leaky gut, microbial translocation, Whipple's disease

## Abstract

**Background & Aims:**

Classical Whipple's disease (CWD) affects the gastrointestinal tract and causes chronic diarrhea, malabsorption, and barrier dysfunction with microbial translocation (MT). Immune reconstitution inflammatory syndrome (IRIS) is a serious complication during antimicrobial treatment of CWD. The pathomechanisms of IRIS have not been identified and mucosal barrier integrity has not been studied in patients with IRIS CWD.

**Methods:**

In 96 CWD patients (*n* = 23 IRIS, *n* = 73 non‐IRIS) and 30 control subjects, we analysed duodenal morphology by histology, measured serum markers of MT, and proinflammatory cytokines in biopsy supernatants, and correlated microbial translocation with T cell reconstitution and activation.

**Results:**

Before treatment, duodenal specimens from patients who later developed IRIS exhibited a more pronounced morphological transformation that suggested a disturbed barrier integrity when compared with the non‐IRIS group. Villous atrophy was mediated by increased apoptosis of epithelial cells, which was insufficiently counterbalanced by regenerative proliferation of crypt cells. Pretreatment deficiencies in the mucosal secretion of proinflammatory cytokines and chemokines (e.g., IL‐6, CCL2) in these patients markedly resolved after therapy induction. High serum levels of lipopolysaccharides (LPS), soluble CD14 (sCD14), and LPS‐binding protein (LBP) combined with low endotoxin core antibody (EndoCAb) titres suggested systemic MT in CWD patients developing IRIS. CD4^+^ T cell count and activation in IRIS CWD patients correlated positively with sCD14 levels and negatively with EndoCAb titres. Furthermore, the degree of intestinal barrier dysfunction and MT was predictive for the onset of IRIS.

**Conclusion:**

Prolonged MT across a dysfunctional intestinal mucosal barrier due to severe tissue damage favors dysbalanced immune reconstitution and systemic immune activation in IRIS CWD. Therefore, the monitoring of inflammatory and MT markers in CWD patients might be helpful in identifying patients who are at risk of developing IRIS. Therapeutic strategies to reconstitute the mucosal barrier and control inflammation could assist in the prevention of IRIS.

## Graphical abstract

Prolonged microbial translocation across a dysfunctional intestinal mucosal barrier due to severe tissue damage favors dysbalanced immune reconstitution and systemic immune activation in Whipple's disease immune reconstitution inflammatory syndrome.

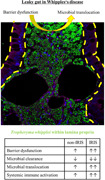

## 1 INTRODUCTION

Classical Whipple's disease (CWD) is caused by infection with *Tropheryma whipplei*. Gastrointestinal symptoms in this disease, such as chronic diarrhea and malabsorption, clearly indicate a dysfunction of the small intestinal mucosa.^1^, ^2^

During antimicrobial treatment, up to 10% of CWD patients are affected by immune reconstitution inflammatory syndrome (IRIS).^3^ The occurrence of IRIS CWD is associated with a rapid and severe clinical deterioration that results in significant morbidity and mortality.^3^, ^4^

IRIS, which was first described in cases of human immunodeficiency virus (HIV) infection, occurs as a paradoxical worsening of pre‐existing infectious processes after induction of antiretroviral therapy (ART) and is characterized by a rapid, dysbalanced restoration of immune function.^5^ Initial clinical improvement is followed by significant deterioration marked by CD4^+^ T cell reconstitution (numerical and/or functional) paralleled by an increased level of inflammation.^6^

It has been hypothesized that HIV‐related mycobacterial IRIS is mediated by a temporal uncoupling of the innate and adaptative immune response.^6^, ^7^ Primed but incompletely activated macrophages (due to a lack of CD4^+^ secondary signal for myeloid activation) in the immunosuppressed host lead to a significant increase in the mycobacterial burden.^6^, ^7^ T cell reconstitution associated with ART initiation then triggers secondary myeloid activation of the accumulated mycobacterium‐primed macrophages.^6^, ^7^ This hyperactivation is associated with a subsequent cytokine storm leading to the tissue damage observed in IRIS.^6^, ^7^

The pathognomonic hallmark of CWD (both in IRIS and non‐IRIS patients) is massive infiltration of the small intestinal lamina propria (LP) with *T. whipplei*‐infected macrophages.^1^ The lack of adequate local inflammation and alternative activation of macrophages leads to insufficient degradation of *T. whipplei* and its systemic spread.^8^, ^9^, ^10^ Treatment induction is associated with immune reconstitution, both in IRIS and non‐IRIS patients^4^; however, in IRIS CWD, after initial improvement with effective antimicrobial therapy, the inflammation reappears.^4^ IRIS CWD is mediated by nonspecific activation of CD4^+^ T cells that is not sufficiently counterbalanced by regulatory T cells (T_regs_).^4^ The pathomechanisms of exacerbated T cell activation in IRIS CWD are still unclear.

It has been shown that CWD patients have a dysfunctional mucosal barrier, provoking diarrhea and increased microbial translocation (nonphysiological passage of gastrointestinal microflora through the intestinal epithelial barrier), and resulting in systemic immune activation.^2^ An increase in epithelial permeability with subsequent microbial translocation and immune stimulation could, therefore, be a potential mediator in the pathophysiology of IRIS.^11^, ^12^, ^13^

The aim of the present study was to assess small intestinal barrier function and bacterial translocation and to correlate it with markers of enhanced immune activation in IRIS CWD.

## 2 MATERIALS AND METHODS

### 2.1 Study design

For all patients, initial blood and tissue samples were obtained at the time of diagnosis, before initiation of antimicrobial treatment for CWD. Depending on the course of their disease, patients were retrospectively allocated to the group of CWD patients not developing IRIS (non‐IRIS CWD patients) or to the group of CWD patients developing IRIS (IRIS CWD patients). Patient reassessment was carried out as symptoms diminished, up to 3 years after the initiation of antimicrobial therapy (non‐IRIS CWD patients—treated), when IRIS became active (active IRIS), and after successful treatment of IRIS (IRIS CWD patients—after IRIS treatment). Successful *T. whipplei*‐directed antimicrobial treatment of all patients was confirmed by a negative tissue PCR and the histological score of duodenal biopsies (both for *T. whipplei*).^3^ Data sets from CWD patients were compared to healthy subjects without gastrointestinal symptoms and patients suffering from acute infectious gastroenteritis (obtained during an enterohemorrhagic *Escherichia coli* (EHEC) serotype O104:H4 outbreak in 2011 in Germany).^14^

### 2.2 Study population

Blood and/or biopsy samples from 73 non‐IRIS CWD patients, 23 CWD patients developing IRIS, and 30 control subjects were collected (for details, see Table 1). All CWD patients exhibited gastrointestinal symptoms at the time of diagnosis, which was confirmed by periodic acid‐Schiff (PAS) staining or *T. whipplei*‐specific antibody staining and tissue PCR.^1^ Antimicrobial treatment was initiated in most cases with intravenous ceftriaxone for 2 weeks, followed by 3 or 12 months of oral trimethoprim‐sulfamethoxazole. In 11 patients, treatment was started with intravenous meropenem, 2 patients received only trimethoprim‐sulfamethoxazole, and 4 patients were alternatively treated with doxycycline in combination with hydroxychloroquine.

**Table 1:** Investigated samples from patients with/without IRIS and controls

	IRIS CWD patients	Non‐IRIS CWD patients	Control subjects
Total	Samples from 23 patients 5 F, 18 M; 55.8 y (42–75 y)	Samples from 73 patients 14 F, 59 M; 57.3 y (37–82 y)	Samples from 30 control subjects: 20 healthy subjects 8 F, 12 M; 48.9 y (24–90 y); 10 subjects with acute infectious enteritis 6 F, 4 M^a^; 48.5 y (26–73 y)
Blood	Samples from 19 patients 4 F, 15 M; 55 y (42–70 y)	Samples from 61 patients 12 F, 49 M; 56.9 y (37–82 y)	Samples from 20 control subjects: 10 healthy subjects 5 F, 5 M; 47.8 y (24–90 y); 10 subjects with acute infectious enteritis 6 F, 4 M^a^; 48.5 y(26–73 y)
Duodenal biopsies	Samples from 16 patients 4 F, 12 M; 56.4 y (43–75 y)	Samples from 39 patients 6 F, 33 M; 57 y (43–74 y)	Samples from 10 healthy subjects 3 F, 7 M; 52.4 y (24–83 y)

*Note*: F: female, M: male; y: years; *p* value (age, sex) >.05.

Abbreviations: CWD, classical Whipple's disease; IRIS, immune reconstitution inflammatory syndrome.

^a^ *p* value < .05 (sex) for non‐IRIS CWD versus acute infectious enteritis.

A detailed description of the IRIS patients' clinical presentations is given in two previous publications.^3^, ^4^ The most common presentations were fever (*n* = 17), lung affection (*n* = 5), CNS symptoms (*n* = 5), recurrent arthritis (*n* = 5), inflammatory ocular/orbital manifestations (*n* = 5), skin lesions (*n* = 4), and intestinal involvement (two patients had small bowel perforation). All patients received steroid therapy when IRIS manifested.^3^

Peripheral blood was collected in sodium‐heparinised and serum tubes (Vacutainer; BD Biosciences) and processed within 24 h. Plasma and serum were stored in polypropylene tubes at −80°C until use. Duodenal biopsy specimens were obtained by routine endoscopy from CWD patients and from control subjects who did not have any pathological findings during endoscopy for cancer screening or dyspeptic problems. Duodenal biopsies were either cultured for assessment of cytokine production or fixed in 4% paraformaldehyde (Sigma) and embedded in paraffin for immunohistochemical analysis.

### 2.3 Short‐term culture of intestinal biopsy samples

Culture supernatants of duodenal biopsies were prepared as described in another study.^15^ Immediately after endoscopy, biopsies were placed into phosphate‐buffered saline (PAA Laboratories), washed, weighed, and incubated on metal mesh covered with RPMI 1640 medium (Invitrogen) containing 10% fetal calf serum (Sigma), 100 U/ml penicillin, 100 µg/ml streptomycin, and 2.5 µg/ml amphotericin (Biochrom) on a shaking platform for 48 h at 37°C in a humidified 5% CO_2_/80% O_2_ atmosphere. Supernatants were stored at −80°C until assay.

### 2.4 Systemic and duodenal cytokine secretion

Concentrations of IL‐6, CCL2, CCL5, and CX3CL1 in supernatants of biopsy cultures were quantified by BD^TM^ cytometric bead array (BD Biosciences), according to the manufacturer's protocol.

### 2.5 Markers of microbial translocation

Endotoxin core antibody (EndoCAb)‐enzyme‐linked immunoassay (ELISA) detects the presence of antibodies against the inner core of endotoxin rough‐lipopolysaccharides (LPS) from four Gram‐negative bacterial species. EndoCAbs, LPS‐binding protein (LBP), and soluble CD14 (sCD14) were analysed by ELISA according to the manufacturers' protocol (Hycult Biotech). LPS were detected with the limulus amebocyte lysate chromogenic end‐point assay (Charles River Laboratories).

### 2.6 Histology, immunohistochemistry, and morphometry

Immunostaining was performed on paraffin sections of duodenum, as described previously.^8^ Primary rabbit antibodies against cleaved caspase‐3 (Asp175) (Cell Signaling Technology) and mouse IgG1 against Ki‐67 (clone MIB‐1; DakoCytomation) were detected by biotin‐conjugated donkey anti‐rabbit F(ab')_2_ fragment, donkey anti‐mouse IgG (both Jackson ImmunoResearch), and streptavidin−alkaline phosphatase (Sigma), and visualized using Fast Red (DakoCytomation). Primary antibodies against caspase‐3, Ki‐67, and *T. whipplei* were incubated for 1 h at room temperature (20°C). Nuclei were counterstained with Mayer's Hematoxylin (DakoCytomation).

For each specimen, 10 villi and 30 contiguous crypt spaces were analysed for active caspase or Ki‐67‐positive enterocytes. The crypt‐villus junction was defined as described by Holt et al.^16^ Goblet cell‐, apoptotic‐, and proliferation‐indexes are expressed as percentages of the total amount of epithelial cells per crypt/villus. Mean villus height was determined from five villi oriented in a sagittal plane per subject using ImageJ‐software (NIH).

### 2.7 FACS analysis of absolute cell numbers in the peripheral blood

T cell numbers from untreated and treated patients, that have been previously published,^4^ were determined using CD3/CD4/CD8 TriTest and mouse anti‐human CD25 (clone 2A3; all from BD Biosciences) according to the manufacturer's protocol. CD25^+^CD4^+^ T cells minus CD25^high^CD4^+^ T cells were designated as activated T cells.^4^ Data acquired on a FACSCalibur or a FACS Canto II device were analysed with CellQuest (all from BD Biosciences) and FlowJo (Tree Star) software.^4^

### 2.8 Statistical analysis

The values for villus length, apoptosis, proliferation, LPS, LBP, and sCD14 in non‐IRIS CWD patients and controls, as well as T cell counts in IRIS CWD patients, have been previously published.^2^, ^4^, ^9^ After performing normality testing, single comparisons were assessed using an unadjusted, unpaired two‐tailed the Student *t* test. Differences among groups were analysed with analysis of variance followed by the Bonferroni‐adjusted *t* test. For correlation analysis, the Spearman or Pearson coefficient was used. Kaplan–Meier analysis of tertiles with a log‐rank Mantel–Cox test was used to calculate the probability of developing IRIS. All analyses were performed using GraphPad Prism version 9.2.0 software. Results are expressed as single values ± standard deviation. The overall α‐level was .05.

## 3 RESULTS

### 3.1 Sustained disruption of mucosal and epithelial integrity in the duodenum in IRIS CWD

**Figure 1:**
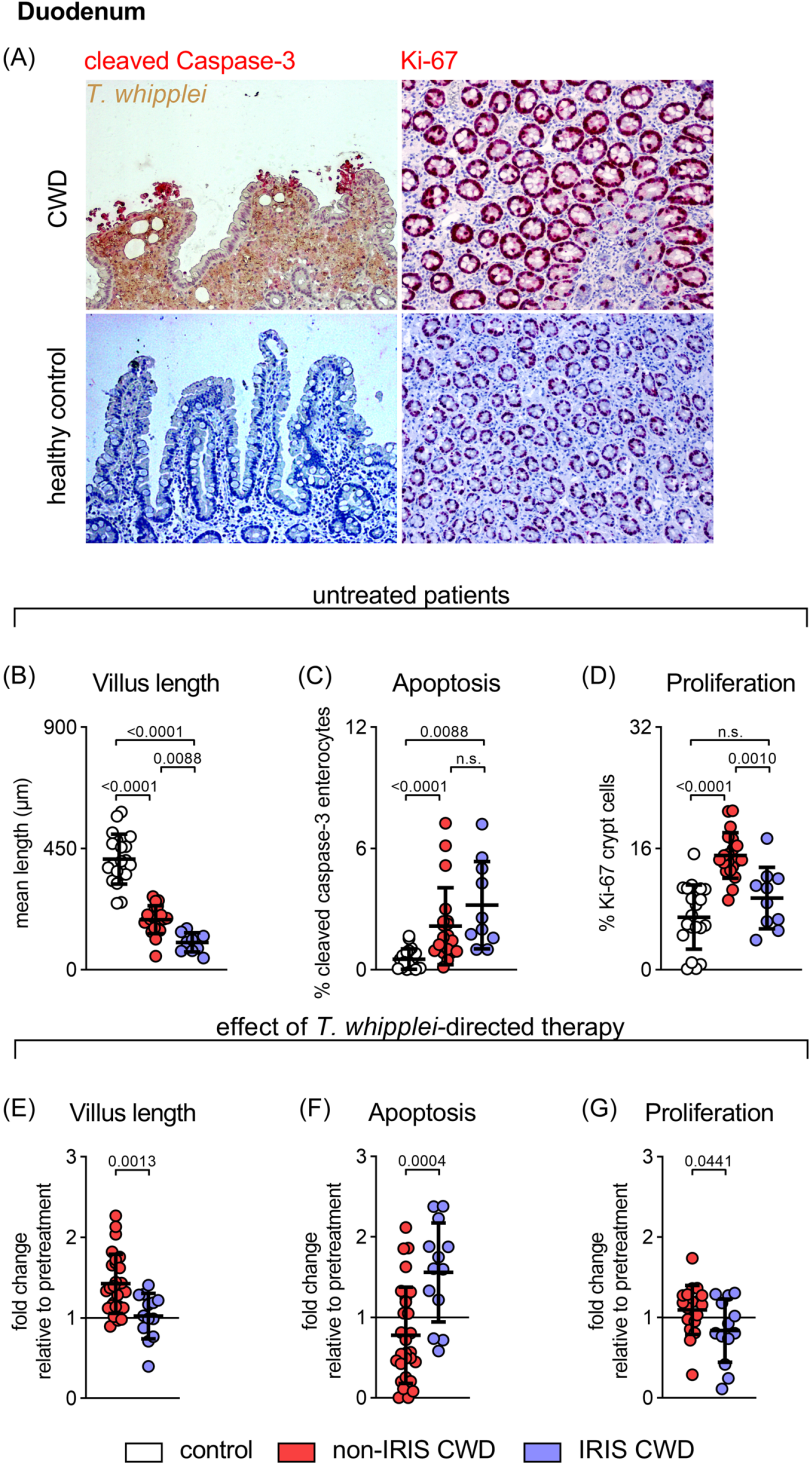
IRIS CWD is characterized by initial distinct and sustained mucosal transformation. (A) Representative sections of duodenal mucosa obtained from untreated patients with CWD (upper panel) and healthy controls (lower panel). Massive infiltration of the small intestinal LP with *T. whipplei*‐infected macrophages (*T. whipplei‐*specific antibody, brown). Apoptotic cells visualized by caspase‐3 staining (red). Proliferating cells visualized by Ki‐67 staining (red). (B–G) Histological examination of duodenal biopsies from non‐IRIS CWD (red circles) and IRIS CWD patients (blue circles) before (B–D) and after (E–G) *T. whipplei*‐directed treatment in comparison to healthy controls (white circles). (B) Morphometric changes in villus architecture expressed as mean villus length. (C) Apoptotic index indicated by active caspase‐3 staining. (D) Proliferating crypt cells indicated by Ki‐67 staining. (E–G) Disease progression in IRIS CWD patients compared to non‐IRIS CWD patients after initiation of *T. whipplei*‐directed therapy (fold change before vs. after treatment; black line indicates no change), with sustained villous atrophy (E), increase in epithelial apoptosis (F), and reduced regenerative proliferation within crypts (G). Comparative values of healthy controls and non‐IRIS CWD patients have previously been published.^2^ Results are expressed as single values (control *n* = 20, non‐IRIS CWD *n* = 19–28, IRIS CWD *n* = 10–13), mean ± *SD*. n.s. = nonsignificant. ANOVA followed by the Bonferroni‐adjusted *t* test (B–D), unadjusted, unpaired two‐tailed Student's *t* test (E–G). ANOVA, analysis of variance; CWD, classical Whipple's disease; IRIS, immune reconstitution inflammatory syndrome

The pathognomonic hallmark of CWD (in both IRIS and non‐IRIS patients) is massive infiltration of the small intestinal LP with *T. whipplei*‐infected macrophages (Figure 1A). The duodenal architecture in patients with CWD is characterized by villous atrophy (Figure 1A).^2^ Mucosal transformation in CWD mediates gastrointestinal symptoms such as chronic diarrhea and malabsorption.^2^

Marked villous atrophy, as compared with healthy subjects and non‐IRIS CWD patients, was indicated by more pronounced villus length reduction in CWD patients who later developed IRIS (mean villus length 54% shorter than healthy controls, 45% shorter than non‐IRIS CWD patients) (Figure 1B). Following initiation of *T. whipplei‐*directed antimicrobial treatment, villus length was restored in CWD patients, but remained reduced in IRIS CWD patients when compared with non‐IRIS CWD patients (Figure 1E).

Mucosal architecture depends on the balance between apoptosis of mature surface enterocytes and proliferation of undifferentiated enterocytes within the crypts.^2^, ^17^ We determined the extent of regenerative turnover by staining the intestinal epithelium for markers of apoptosis and proliferation. When compared with healthy controls and non‐IRIS CWD patients, patients who later developed IRIS had an elevated initial apoptotic index in the villus compartment, as determined by active caspase‐3 staining (Figure 1C). After treatment induction, epithelial apoptosis increased in IRIS CWD patients when compared with non‐IRIS patients who had decreased villous cell loss (Figure 1F).

Mucosal damage mediated by excessive epithelial cell loss was not sufficiently counterbalanced by Ki‐67^+^ proliferating crypt cells in IRIS CWD (Figure 1D). Furthermore, CWD patients who later develop IRIS exhibited a lower regenerative potential of their small intestinal mucosa during the disease course, as indicated by an increased number of epithelial cells that underwent apoptosis and reinforced by a sustained low rate of proliferation within the crypts (Figure 1G). This more pronounced epithelial damage in IRIS CWD patients would allow for increased epithelial passage of microbial‐ and food‐derived macromolecular components.^2^

### 3.2 Deficiency of duodenal proinflammatory cytokines and chemokines in untreated IRIS CWD patients and their reconstitution after therapy induction

**Figure 2:**
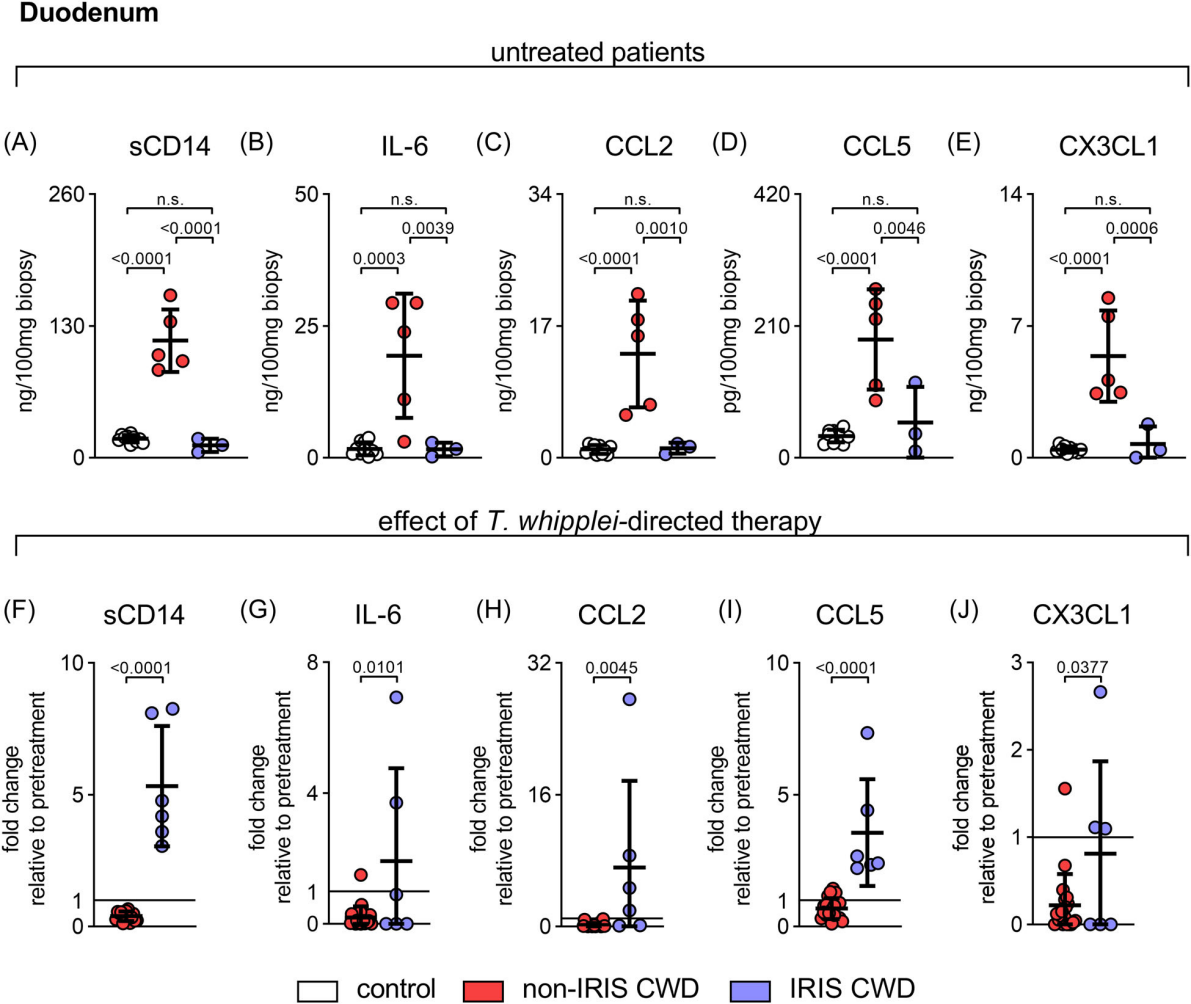
Deficiency of duodenal proinflammatory cytokines and chemokines in untreated IRIS CWD patients and their induction after therapyinitiation. Proinflammatory innate chemokines and cytokines were determined in supernatants of cultured duodenal biopsies from untreated (upper panel, A–E) and treated (lower panel, F–J) CWD patients. (F–J) Disease progression in IRIS CWD patients compared to non‐IRIS CWD patients after induction of *T. whipplei*‐directed therapy (fold change before vs. after treatment; black line indicates 100% [no change]). Results are expressed as single values (control *n* = 10, non‐IRIS CWD *n* = 5–20, IRIS CWD *n* = 3–6), mean ± *SD*. n.s. = nonsignificant. ANOVA followed by the Bonferroni‐adjusted *t* test (A–E), unadjusted, unpaired two‐tailed the Student *t* test (F–J). ANOVA, analysis of variance; CWD, classical Whipple's disease; IRIS, immune reconstitution inflammatory syndrome

A proinflammatory innate immune response within the LP would be the first line of defense against increased microbial translocation from the gut lumen.^18^ However, in contrast to untreated non‐IRIS CWD patients, the patients who later developed IRIS initially exhibited a marked reduction in proinflammatory chemokines and cytokines within the duodenum (Figure 2A–E).

In contrast to non‐IRIS CWD, the proinflammatory innate immune response in the small intestine recovered during IRIS CWD (Figure 2F–J), which may promote local tissue injury and prolong barrier leakage.

### 3.3 Biomarkers suggest distinct and sustained elevation of circulating microbial products

Elevated levels of LPS, sCD14, and LBP and decreased levels of LPS‐neutralizing EndoCAbs in serum/plasma are well defined surrogate markers of increased intestinal permeability and microbial translocation.^19^, ^20^, ^21^, ^22^, ^23^, ^24^ In non‐IRIS CWD patients, increased serum markers of microbial translocation and their decline following treatment corroborated the biological significance of the mucosal barrier defect.^2^ LPS is the prototype of a gut‐derived, translocated microbial product. Whereas sCD14 and LBP are elevated in response to endotoxemia, LPS‐neutralizing EndoCAbs are consumed and are thus reduced in enhanced endotoxin loads.^20^, ^25^, ^26^

Mucosal damage, as indicated by an increased apoptotic index in the villus compartment, was associated with elevated plasma levels of sCD14 (Pearson *r* = .7384, *p* < .0001) and inversely correlated with EndoCAb titres (Pearson *r* = −.5364, *p* < .0083) in IRIS CWD patients.

**Figure 3:**
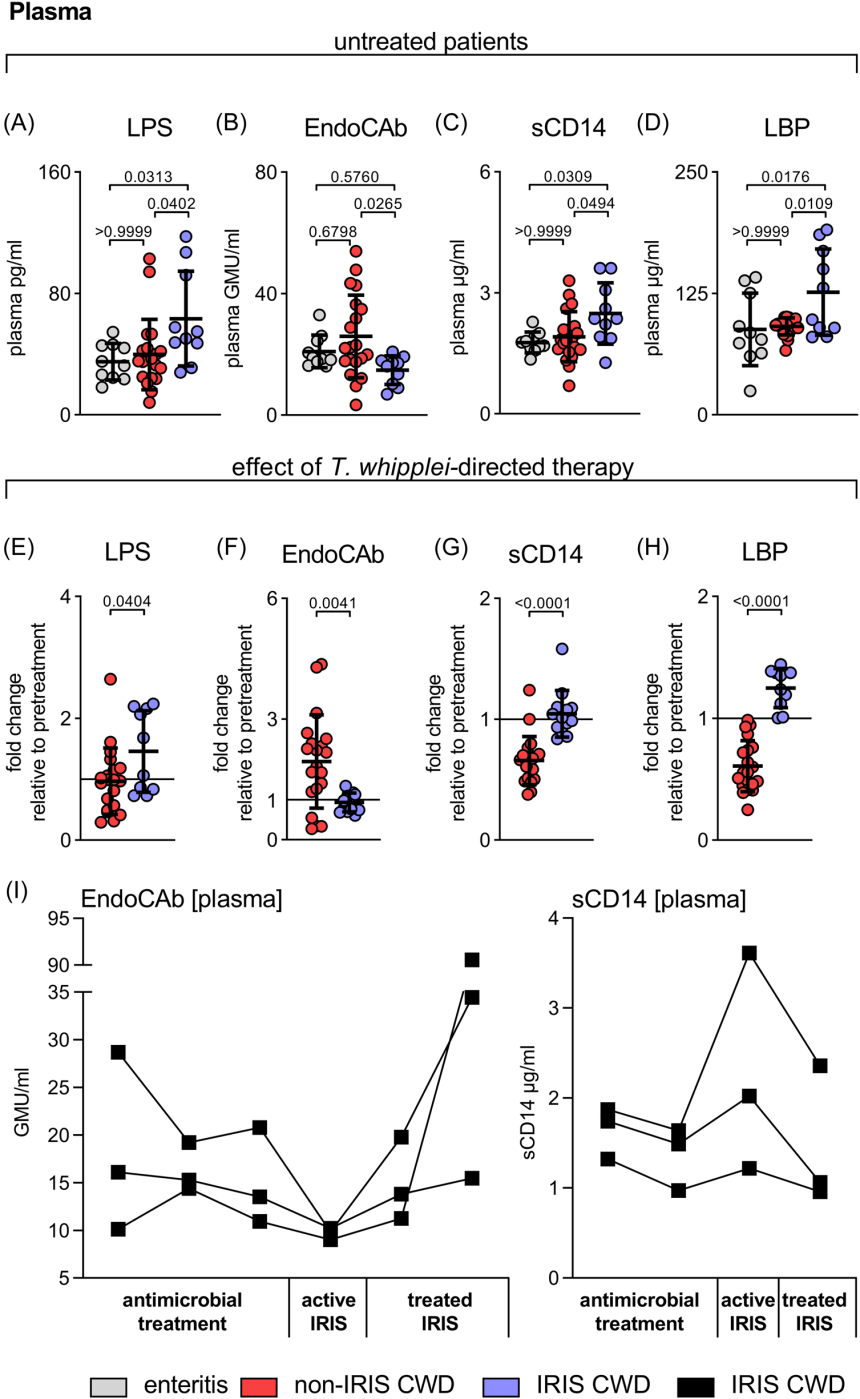
Biomarkers suggest distinct and sustained elevation of circulating microbial products in IRIS CWD. Concentrations of markers for increased microbial translocation in plasma was measured by ELISA. (A–D) LPS, EndoCAb (IgG), sCD14, and LBP in plasma samples from untreated non‐IRIS and IRIS CWD patients compared to patients with acute infectious gastroenteritis (grey circles). (E–H) Disease progression in IRIS CWD patients compared to non‐IRIS CWD patients after induction of *T. whipplei*‐directed therapy (fold change before vs. after treatment; black line indicates no change). Comparative values of LPS, sCD14, and LBP from non‐IRIS CWD patients have previously been published.^2^ Results are expressed as single values (enteritis *n* = 10, non‐IRIS CWD *n* = 20, IRIS CWD *n* = 10–13), mean ± *SD*. n.s. = nonsignificant. ANOVA followed by the Bonferroni‐adjusted *t* test (A–D), unadjusted, unpaired two‐tailed Student's *t* test (E–H). (I) Changes in EndoCAb and sCD14 levels in three CWD patients developing IRIS for which consecutive sampling was available. ANOVA, analysis of variance; CWD, classical Whipple's disease; ELISA, enzyme‐linked immunoassay; IgG, immunoglobulin G; IRIS, immune reconstitution inflammatory syndrome

Compared to non‐IRIS CWD patients, LPS levels were initially higher in the IRIS group (Figure 3A). In CWD patients developing IRIS, EndoCAb titres were lower than those of non‐IRIS CWD patients and similar to those of patients with acute infectious enteritis‐associated gut‐derived microbial translocation (Figure 3B).^25^ In the non‐IRIS group, EndoCAb titres increased directly after induction of *T. whipplei*‐directed therapy (Figure 3F). In contrast, in CWD patients who developed IRIS, EndoCAb titres remained low and did not increase until specific therapy for IRIS was initiated (Figure 3F and 3I).

In line with these observations, patients who later developed IRIS had initially higher levels of sCD14 and LBP that persisted even after induction of *T. whipplei*‐directed therapy, as compared with non‐IRIS CWD patients (Figure 3C,D and 3G–I).

These results suggest a sustained barrier dysfunction and microbial translocation in CWD patients who later develop IRIS.

### 3.4 Biomarkers that are suggestive for increased microbial translocation, correlate with T cell reconstitution and activation in patients with IRIS CWD

We have demonstrated that, in CWD patients who later develop IRIS, surrogate marker of barrier dysfunction and microbial translocation are initially enhanced compared with non‐IRIS CWD patients, and these processes persist despite *T. whipplei*‐directed therapy induction. Because IRIS CWD is mediated by uncontrolled T cell restoration,^4^ we questioned whether microbial translocation might be linked to T cell reconstitution and activation in IRIS CWD.

**Figure 4:**
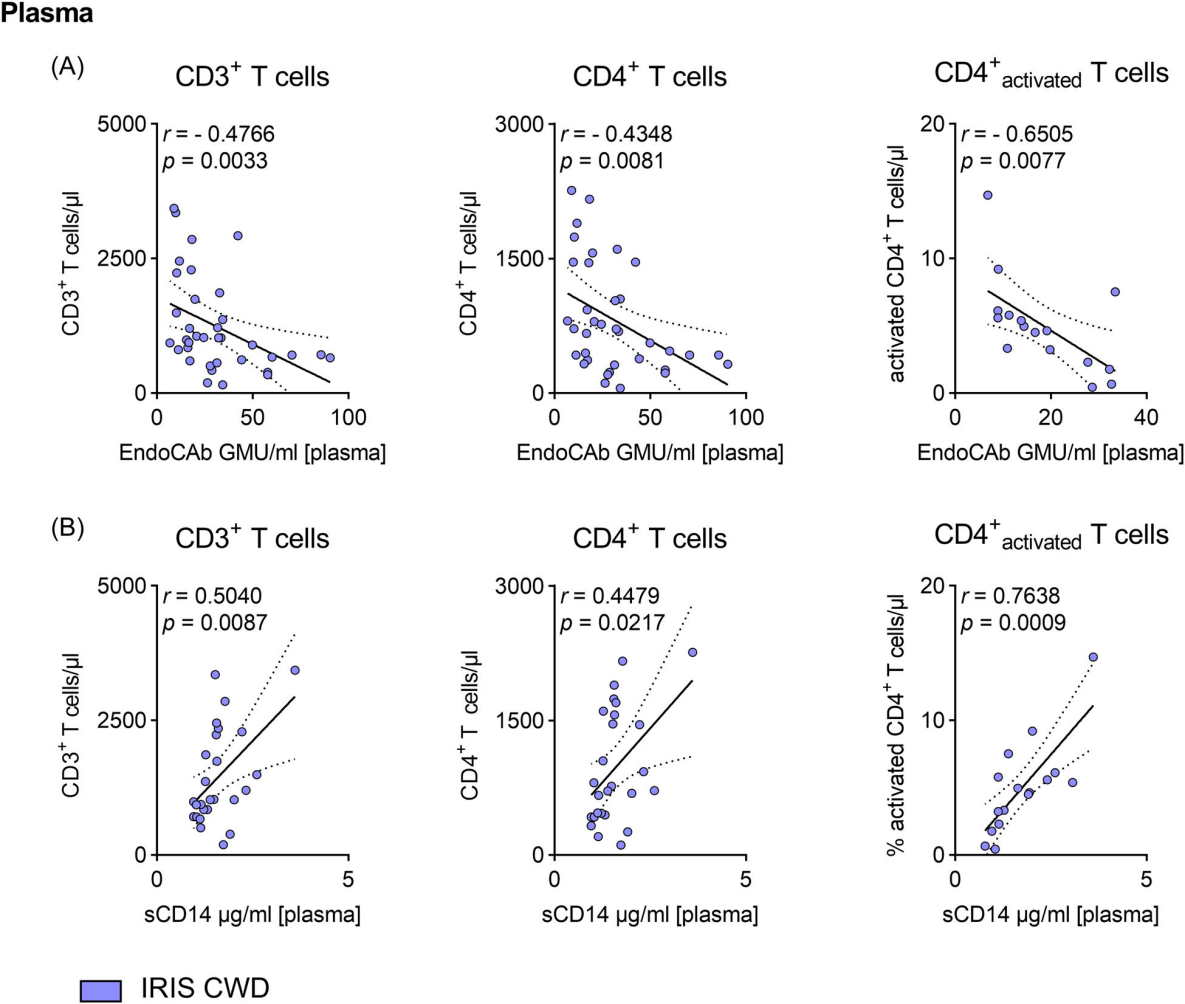
Biomarkers that are suggestive for increased microbial translocation, correlate with T cell reconstitution and activation in patients with IRIS CWD. Plasma levels of EndoCAbs (A) and sCD14 (B) relative to the circulating number of CD3^+^ T cells, CD4^+^ T cells, and percentage of activated CD4^+^ T cells. CD25^+^CD4^+^ T cells minus CD25^high^CD4^+^ T cells were designated as activated T cells.^4^ Comparative values for T cell counts have previously been published.^4^ Results are expressed as single values (*n* = 16–36 of untreated and treated patients), Spearman correlation coefficients, and linear regression lines with 95% CI. CI, confidence interval

Therefore, we correlated markers of microbial translocation with peripheral T cell counts. Low EndoCAb titres were associated with high CD3^+^ and CD4^+^ T cell count and inversely correlated with the number of circulating activated CD4^+^ T cells (Figure 4A). In line with these observations, plasma levels of sCD14 positively correlated with the number of CD3^+^, CD4^+^, and activated CD4^+^ T cells (Figure 4B).

These data highlight the connection between a leaky gut and the inflammation triggered by the uncontrolled reconstituted T cell compartment.

### 3.5 Surrogate marker of intestinal barrier dysfunction and microbial translocation correspond to the probability of developing IRIS in patients with CWD

**Figure 5:**
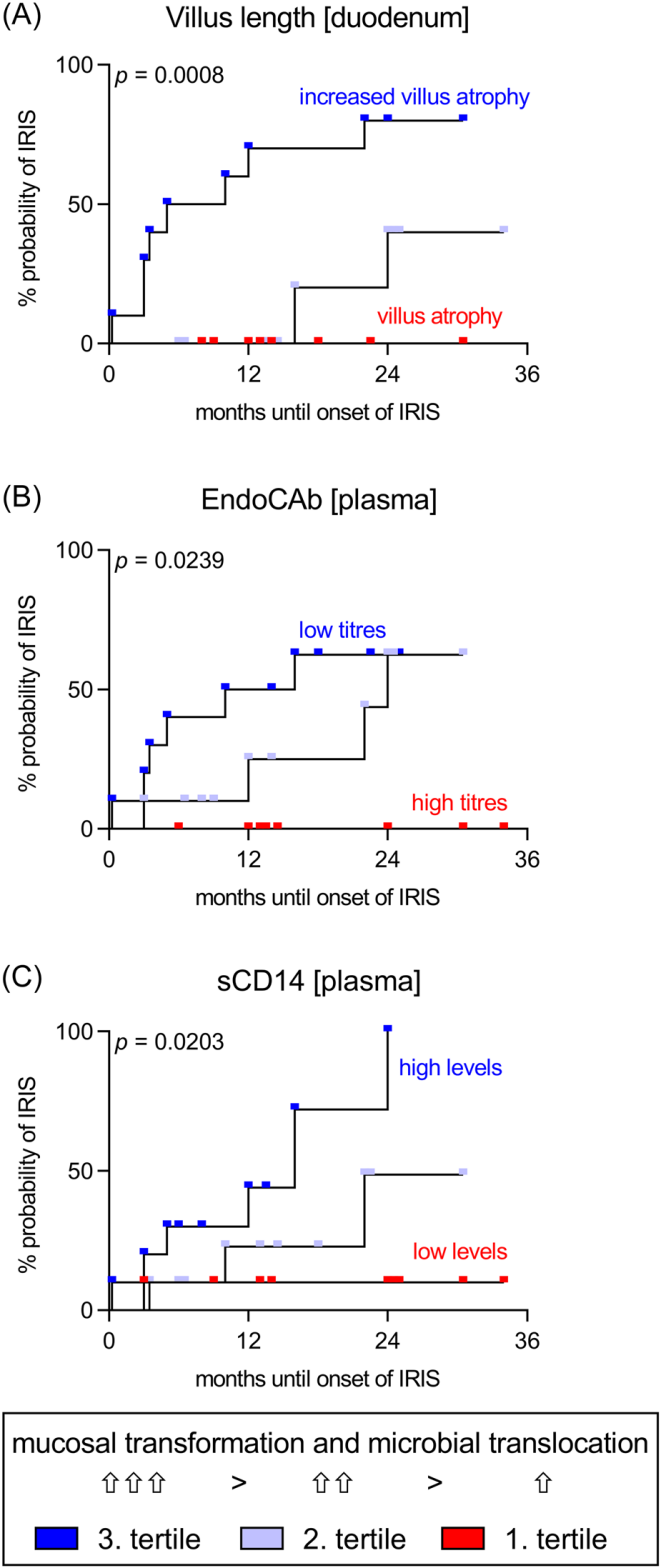
Surrogate marker of intestinal barrier dysfunction and microbial translocation correspond to the probability of developing IRIS in patients with CWD. Kaplan–Meier curves for tertiles of indirect indicators of barrier dysfunction (mean villus length, (A) and elevated biomarker of microbial translocation (EndoCAb, B; sCD14, C) in untreated CWD patients (*n* = 30; 20 non‐IRIS CWD, 10 IRIS CWD). For each indicator, the groups were independently organized into tertiles (*n* = 10) and assessed as independent predictors of developing IRIS after induction of *T. whipplei*‐directed antimicrobial therapy. Mean villus length: 190–272 µm, 140–189 µm, and 43–139 µm. EndoCAb: 22.1–54 GMU/ml, 17–22 GMU/ml, and 3–16.9 GMU/ml. sCD14: 0.7–1.8 µg/ml, 1.81–2.3 µg/ml, and 2.31–3.7 µg/ml. The *p* value indicates the significance of the log‐rank Mantel–Cox test. CWD, classical Whipple's disease; IRIS, immune reconstitution inflammatory syndrome

The association between the onset of IRIS and indirect indicators of intestinal barrier dysfunction and microbial translocation in untreated CWD patients was tested by Kaplan–Meier analysis of tertiles. The degree of mucosal transformation and putative barrier disturbance (as indicated by villous atrophy) and the burden of microbial translocation (as suggested by consumption of EndoCAbs and elevation of sCD14) corresponded to the probability of developing IRIS after induction of *T. whipplei*‐directed therapy (Figure 5).

## 4 DISCUSSION

Immunopathology in CWD is characterized by the dichotomy of immunoregulatory mechanisms and features of systemic immune activation.^8^, ^9^, ^10^, ^27^ In non‐IRIS CWD patients, gut homeostasis reconstitutes and inflammatory processes resolve after initiation of antimicrobial therapy, whereas recurrent inflammation can be observed in patients who develop IRIS.^4^ Circulating microbial products have been demonstrated to contribute to the immune pathogenesis of inflammatory bowel disease,^23^ HIV infection,^24^ and ART‐associated IRIS in HIV‐infected patients.^7^ Furthermore, compromised gut immunity with increased microbial translocation into the systemic circulation has already been discussed as a mechanism of immune stimulation in IRIS.^11^

In this study, we demonstrated that patients developing IRIS (in contrast to non‐IRIS patients):

1. are characterized by initial distinct and sustained mucosal transformation, suggestive for the disruption of intestinal epithelial barrier integrity,
2. lack duodenal proinflammatory cytokines and chemokines that recover after initiation of therapy,
3. show (directly and indirectly) a distinct and sustained systemic elevation of circulating microbial products, and
4. reveal a correlation between biomarkers suggestive of increased microbial translocation and T cell reconstitution and activation.

An intact epithelial layer and regular mucosal architecture are prerequisites for the maintenance of small intestinal barrier function and homeostasis. In non‐IRIS CWD patients, electrophysiological and flux experiments revealed increased duodenal permeability to small solutes and macromolecules.^2^ The degree of intestinal barrier dysfunction and microbial translocation (measurement of indirect surrogate marker) in our CWD patients was highly predictive for the onset of IRIS. Accordingly, our data suggested that the barrier defects of the intestinal mucosa in IRIS CWD patients was more severe and persistent than in non‐IRIS patients. Naturally, small intestinal integrity is maintained by balanced epithelial cell turnover, while excessive cell death facilitates gut pathology and systemic immune response.^28^ In the context of non‐IRIS CWD, dysregulated cell turnover and an increase in epithelial apoptosis affect the mucosal integrity, as illustrated by villus atrophy that would allow for increased epithelial passage of microbe‐ and food‐derived macromolecular components.^2^ Despite effective treatment, the disruption of epithelial integrity was more distinct and prolonged in CWD patients developing IRIS, which likely promoted local inflammation and mucosal tissue damage. However, a main limitation of our study is the lack of measuring mucosal barrier permeability directly.

Microbial products (in addition to *T. whipplei*) that enter the LP due to impaired barrier function need to be cleared by innate immune cells. *T. whipplei*‐loaded macrophages are the predominant cell type in the duodenal mucosa in CWD.^1^ It was shown that these macrophages have a reduced phagocytic capacity after exposure to *T. whipplei*, thus promoting the persistence of non‐*T. whipplei* bacterial products in the mucosal tissue, even after successful antimicrobial treatment of CWD.^8^ Moreover, an initial hyporesponsive milieu might further impair sufficient bacterial clearance, as indicated in our study by the absence of proinflammatory innate immune processes and reduced CD4^+^ T cell infiltration of duodenal mucosa of the IRIS group compared with the non‐IRIS group.^4^, ^29^

Therefore, a highly dysfunctional small intestinal barrier together with the absence of a sufficient inflammatory reaction (pretreatment) might contribute to dysbalanced immune reconstitution and subsequent inflammation in IRIS CWD.

Consumption and subsequent reduction of EndoCAbs provides indirect evidence for a leaky gut, and sCD14 and LBP that correlate with endotoxaemic episodes^21^, ^22^ are able to neutralize cell‐bound or circulating LPS to prevent excessive immune stimulation.^21^, ^30^, ^31^, ^32^

In our study, we found extremely low concentrations of EndoCAbs and high levels of LPS, sCD14, and LBP in the serum before initiation of treatment and at the time of acute IRIS, indicating a more severe and long‐lasting endotoxaemic episode with subsequent prolonged immune stimulation in the IRIS CWD cohort compared with the non‐IRIS cohort. Similar findings have been described for patients with acute mucosal barrier failure.^20^, ^25^, ^26^

Dysbalanced reconstitution of antigen‐primed, activated T cells and innate immune cells appears to be a major pathophysiological mechanism in IRIS CWD.^5^ Risk factors that were identified as contributing to recurrent inflammation included: low CD4^+^ T cell count (more severe in IRIS CWD patients),^4^ immunosuppression (lack of local inflammation), and a pre‐existing high pathogen load before initiation of antimicrobial treatment (more severe barrier disruption).^5^

CWD patients exhibit a hyporesponsive regulatory cellular and cytokine milieu that inadequately stimulates T cells.^9^ Therefore, an endotoxaemic episode could be considered a potential mediator of dysbalanced T cell reconstitution. Indeed, we found a correlation between markers of microbial translocation and the peripheral T cell count and T cell activation.

Coupling innate with adaptive immunity and reincreasing levels of sCD14, which have been demonstrated to augment T cell proliferation,^33^ might allow for restoration of activated T cells in patients developing IRIS.^4^

However, when IRIS evolves after therapy induction, a rise in mucosal T cells (mostly memory CD4^+^ T cells with a gut‐homing phenotype) insufficiently counterbalanced by T_regs_ ^4^ might mediate prolonged inflammation and tissue damage through activation of sCD14‐secreting myeloid cells.^21^, ^34^ Consistent with these findings, we found an increase in proinflammatory chemokines, cytokines, and an increase in apoptotic epithelial cells in the duodenal mucosa of IRIS CWD patients. These data are supported by the fact that the stimulating capacity and interferon gamma (IFN‐γ) production of T cells in IRIS CWD patients are initially impaired and increase during IRIS.^4^ Therefore, T cell recovery and reintroducing IFN‐γ production might stimulate the innate immune response.

Synergistic activation of innate effector cells by endotoxins and reconstituting T cells ultimately results in the release of proinflammatory cytokines and is assumed to be a central mechanism in the pathogenesis of IRIS.^5^, ^19^ The CWD‐specific regulatory immunophenotype, with a general anti‐inflammatory milieu, enhanced activity of T_regs_, alternative activation of macrophages, and high IL‐10 production interferes with the classical IRIS‐associated cytokine storm. In this study, increasing levels of IL‐6, CCL2, CCL5, CX3CL1, and sCD14 in the duodenal mucosa reflected local immune activation in IRIS CWD.

We observed a marked and sustained small intestinal epithelial transformation. This suggests a barrier defect of the intestinal mucosa and subsequent increased translocation of gut‐derived microbial products in IRIS CWD patients that became evident by the elevation of surrogate serum markers of microbial translocation. The dysregulated innate and adaptive immune responses in IRIS CWD patients contribute to the persistence of microbial products and biomarker of microbial translocation correlated with elevated T cell counts and activation.

We conclude that prolonged microbial translocation due to a leaky intestinal barrier is a pathomechanism that triggers dysbalanced T cell restoration and inflammatory activation in IRIS CWD.

The monitoring of inflammatory and microbial translocation markers in CWD patients might be helpful for identifying patients who are at risk of developing IRIS and prevent misdiagnosing a treatment failure due to the recurrence of inflammation. This intervention must be tested in future trials. Furthermore, therapeutic strategies aimed to reconstitute the mucosal barrier and control exacerbated inflammation could assist in the prevention of IRIS.^2^, ^35^

## AUTHOR CONTRIBUTIONS

**Julian Friebel, Verena Moos, and Thomas Schneider**:designed the study; **Julian Friebel, Katina Schinnerling, Anika Geelhaar‐Karsch, Kristina Allers, and Verena Moos**: performed experiments; **Julian Friebel and Verena Moos**: collected patient samples; **Julian Friebel, Katina Schinnerling, Anika Geelhaar‐Karsch, Kristina Allers, and Verena Moos**: analysed data; **Julian Friebel**: created the figures; **All authors**: discussed and interpreted the data; **Verena Moos, and Thomas Schneider**: supervised the research; **Julian Friebel**: wrote the manuscript; all authors: read and approved the final draft submitted. All authors have read and agreed to the published version of the manuscript.

## CONFLICTS OF INTEREST

The authors declrae no conflicts of interest.

## ETHICS STATEMENT

The local ethics committee approved the study protocols (registry numbers: 229‐17 and EA4/122/10), which were performed in accordance with the ethical principles in the Declaration of Helsinki. Each patient gave written informed consent before participation in the study.

## Data Availability

The data that support the findings of this study are available on request from the corresponding author. The data are not publicly available due to privacy or ethical restrictions.
